# Chemical composition and antifungal effect of hydroalcoholic extract of *Allium tripedale (Tvautv.)* against *Candida* species 

**DOI:** 10.29252/cmm.3.1.6

**Published:** 2017-03

**Authors:** M Shirani, A Samimi, H Kalantari, M Madani, A Kord Zanganeh

**Affiliations:** 1 Department of Pharmacology and Toxicology, School of Pharmacy, Ahvaz Jundishapur University of Medical Sciences, Ahvaz, Iran; 2 Department of Microbiology, Falavarjan Branch, Islamic Azad University, Isfahan, Iran; 3 Department of Pharmaceutics and Nanotechnology Research Center, School of Pharmacy, Ahvaz Jundishapur University of Medical Sciences, Ahvaz, Iran

**Keywords:** *Allium tripedale*, *Candida* species, Candidiasis, GC-MS

## Abstract

**Background and Purpose:**

Treatment of life-threatening fungal infections caused by *Candida* species has become a major problem. *Candida* spp. are the most important causative agents of candidiasis. *Allium tripedale* is a medicinal plant that has been traditionally used to treat infections. In the present study, we aimed to determine the chemical compounds and antimicrobial activity of hydroalcoholic extract of *A. tripedale* against different species of *Candida.*

**Materials and Methods:**

Phytochemical analysis was performed to identify the possible bioactive components of this extract by using gas chromatography and mass spectroscopy (GC-MS). The hydroalcoholic extract of *A. tripedale* were collected. Different concentrations of *A. tripedale* (50, 25, 12.5, and 6.25 mg/ml) were used to evaluate its antifungal activity against *Candida* species (*C. albicans, C. parapsilosis,* and *C. krusei*) using disk diffusion assay.

**Results:**

The GC-MS analysis revealed the presence of 40 different phytoconstituents with peak area; the major compounds were tetracosane, hexadecanoic acid, 1-eicosanol, 1,2-dihydro-pyrido[3,2,1-kl]phenothiazin-3-one, 2-hexadecen-1-ol, and 3,7,11,15-tetramethyl. Hydroalcoholic extract showed strong antimicrobial activity (inhibition zone ⩾ 20 mm), moderate antimicrobial activity (inhibition zone < 12-20 mm), and no inhibition (zone < 12 mm). In addition, the hydroalcoholic extract exhibited the highest antimicrobial properties against *C. albicans* strains.

**Conclusion:**

*A. tripedale* extract had a considerable inhibitory effect against various *Candida* species, but its highest inhibitory effect was against *Candid albicans. *Further investigations are required to detect the performance of this plant in the treatment of *Candida *infection.

## Introduction

Plants are a great source of useful phytochemicals, which have inhibitory effects against some microorganisms in vitro and are effective in the treatment of various conditions [1]. Generally, 1-10% of plants (out of approximately 250,000-500,000 species) on earth are used by humans [2]. In recent years, there has been a growing global interest in the use of medicinal plants for disease prevention and treatment, especially in Iran [3]. Limited success in the treatment of human diseases, undesirable side effects of chemical drugs, and growing emergence of drug resistance, particularly to antibiotics, have led to increased use of medicinal plants [4]. 

Medicinal plants are a widespread source of biologically active compounds including alkaloids, tannins, flavonoids, and phenolic compounds. Accordingly, they are of marked significance to the health of individuals and communities and are widely used for disease treatment [2]. 

*A. tripedale *belonging to the Liliaceae family*, *is a wild *Allium* species native to the Caucasus (North + South), Iraq, Turkey, and Iran. This plant has longand strong stems (50-90 cm in length) and some what unpleasant taste [5, 6]. *A. tripedale* has been extensively used by locals as a spicy vegetable and for the treatment of infections. Given the presence of saponins in the structure of this plant, it is expected to have inhibitory effect against pathogenic fungi [7].

Since the early 1990s, the increase in the number of infections caused by pathogenic and opportunistic fungi has been introduced as the leading cause of mortality among hospitalized patients [8]. In other words, a large number of people are suffering from fungal infections, and these infections are posing a great threat to mankind [9]. In addition, the increased use of antifungal agents has led to the development of resistance to the available drugs. 

*Candida albicans* as an opportunistic pathogen plays an important role in the infection and is the most common cause of cutaneous, oral, and systemic diseases in immunodeficiency patients [10]. Although *Candida albicans* is still the major species isolated from clinical samples in the majority of individuals,it is well known that some other non*-albicans Candida* spp. such as *Candida glabrata*, *Candida krusei*, *Candida parapsilosis,* and *Candida tropicalis* infections are significantly widespread. Candidiasis associated with this kind of non-*albicans Candida *spp. pose a clinical challenge because they are resistant to common antifungal agents such as fluconazole and amphotericin B [11, 12]. 

Regarding the increase in the use of antifungal agents and resistance to some types of *Candida *spp. and the undesirable side effects of chemical drugs, it is essential to explore new sources of treatment, particulary among herbal plants [8, 13]. To the best of our knowledge, no has yet explored the antifungal activity of *A. tripedale *against *Candida *isolates.The purpose of this study was to evaluate chemical composition and antimicrobial activity of hydroalcoholic extract of *A. tripedale *against different *Candida* spp. 

## Materials and Methods

***Plant collection***

*Tripedale *was collected from the highlands of Shahrekord in southeast of Iran (Isfahan Province). The collected samples were identified in Ahvaz Agricultural and Natural Research Centre (Herbarium No. A151640100AP). Extraction and laboratory examinations were carried out in Ahvaz University of Medical Sciences, Ahvaz, Iran. The aerial parts of the plants were aired indoors at room temperature and then finely powdered using an electric grinder (Busch, MKM6003, Slovenia). It took two days to extract 20 g of plant materials by soxhlet with 120 ml ethanol 80%. The extract was filtered using Whatman qualitative filter paper, Grade 1. The extract was preserved in sterilized airtight bottles at 4ºC, and then to prepare the dried extracts, the solution was placed in a bain-marie at 40ºC for 24 h prior to use [14].

***Gas chromatography and mass spectroscopy (GC-MS) analysis***

GC-MS analysis of ethanolic extract of the whole *A. tripedale* was performed on GC 7890A equipped with MS 5975C detector and HP-5ms capillary column (30 × 0.25 m, 0.25 µm; Agilent Co., USA). The initial column temperature was set at 60ºC, then increased from 60ºC to 190ºC (heating rate: 5ºC per minute), from 190ºC to 270ºC for 30 min, and finally kept at 270ºC for approximately 5 min; the total analysis time was about 34 min. 

***Compound identification***

Interpretation of GC-MS was performed via the National Institute Standard and Technology (NIST) database. The spectra of the unknown components were compared with those of the known ones registered in the NIST library. The name, molecular weight, and structure of the components of the test materials were determined.

***Preparation of organisms***

Standard strains of *C. albicans *(ATCC 3153), *C. parapsilosis* (ATCC 2195), and *C. krusei* (ATCC 573) were obtained from the Department of Mycology, Faculty of Veterinary Medicine, Tehran University, Tehran, Iran. The strains were cultured on Sabouraud Dextrose Agar (SDA) (Merck, Germany) medium. Fungal suspension was prepared with concentration adjusted to 1.5_˟_10^6^ CFU/ml in sterile distilled water as described by Forbes et al. [15].

***Well diffusion assay***

Agar well diffusion method is extensively used to evaluate the antimicrobial activity of plants or microbial extracts. To determine the effective concentration, inhibition zones of hydroalcoholic extract of *A. tripedale* were examined against *C. albicans* (ATCC 3153), *C. parapsilosis* (ATCC 2195), and *C. krusei* (ATCC 573) strains by using well assay technique. The agar plate surface was inoculated overnight by spreading inoculum of *Candida* spp. over the entire SDA surface. A hole 6 to 8 mm in diameter was punched with a sterile tip. Then, the extract was added to the pits in the agar medium and incubated under suitable conditions at 27°C for 24 h [16]. The diameter of the inhibitory zone was measured, and the corresponding effective concentration was chosen for subsequent experiments [17].

***Disk diffusion method***

The fungal broth culture aliquots were added to SDA. Sterile paper disks (Merck, Germany) were impregnated with 50 μl of extract solution and placed on the culture plates. The plates were incubated at 37°C for 24 h. Antifungal activity was evaluated by measuring the inhibition zone diameter [18]. Fluconazole was used as positive control [19], whereas paper disks loaded with solvents (ethanol and distilled water) were used as negative controls. 

***Statistical analysis***

Statistical analysis was performed using SPSS, version 10.0. The inhibition diameters of the test substances were expressed as mean and standard deviation. Group comparisons were performed using One-way analysis of variance (ANOVA) followed by Waller-Duncan Post Hoc test. *P-value* less than 0.05 was considered statistically significant.

## Results

***The phytochemical analysis***

**Figure 1:**
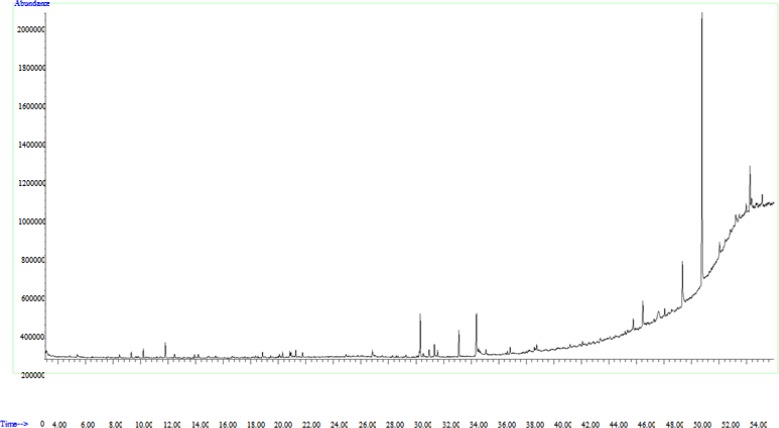
Gas chromatography and mass spectroscopy chromatogram of hydroalcoholic extract of *A. tripedale*

The phytocomponents present in the hydroalcoholic extract of *A. tripedale *were identified by GC-MS analysis; GC-MS running time is 34 min. The active compounds in the hydroalcoholic extract of the plant, their retention time (RT), molecular formula, and molecular weight are provided in Table 1, and GC-MS chromatograms are presented in Figure 1.

The gas chromatogram is used to help identify a mixture of compounds by separating compounds according to each compound's retention time. The heights of the peaks indicate the relative concentrations of the components present in the plant. GC-MS analysis revealed the presence of 40 compounds by dichloromethane solvent; the major compounds included tetracosane, hexadecanoic acid, 1-eicosanol, 1,2-dihydro-pyrido[3,2,1-kl]phenothiazin-3-one, 2-hexadecen-1-ol, and 3,7,11,15-tetramethyl.

***Disk and well diffusion assay***

**Figure 2:**
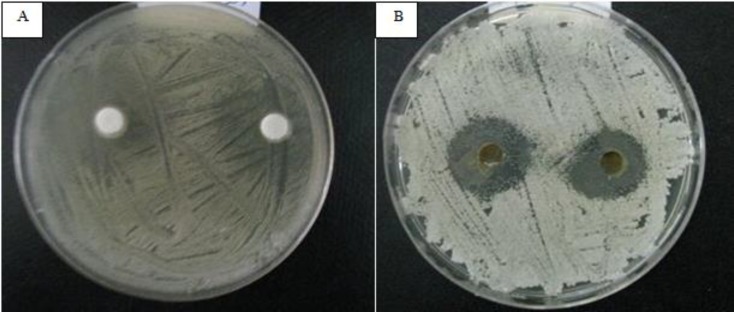
Anti-fungal activity of fluconazole (50 mg/ml) against *C. albicans* by disc (A) and well (B) diffusion assay after 48 h

**Figure 3:**
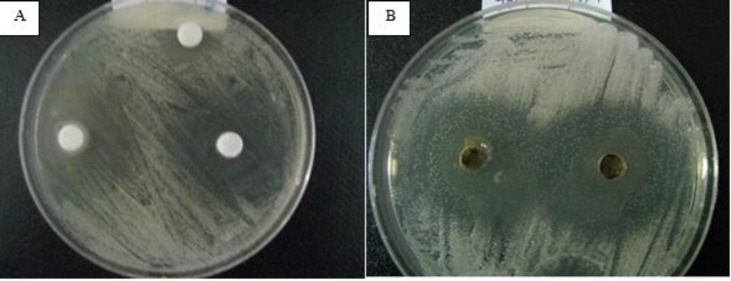
Anti-fungal activity of hydroalcoholic extract of *A. tripedale* (50 mg/ml) against *C. albicans* by disc (A) and well (B) diffusion assay after 48 h

**Figure 4:**
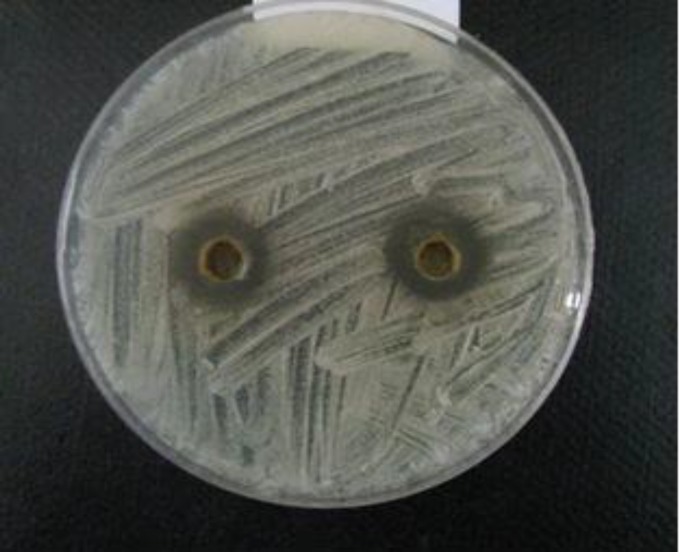
Anti-fungal activity of hydroalcoholic extract of *A. tripedale* (50 mg/ml) against *C. parapsilosis* well diffusion assay after 48 h

**Figure 5:**
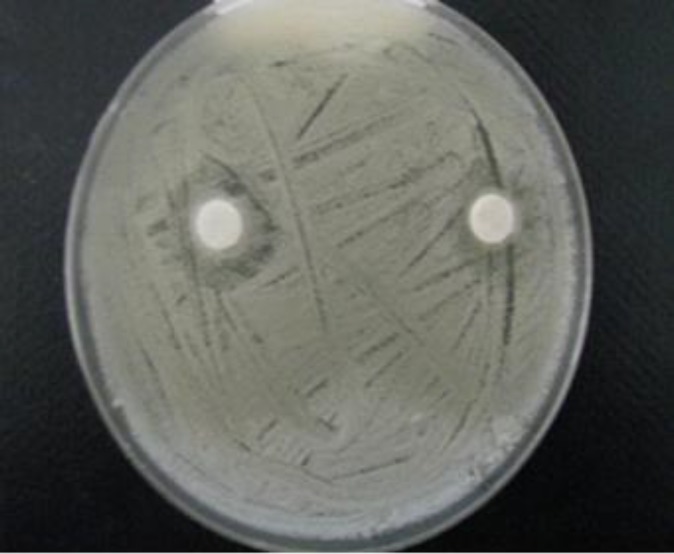
Anti-fungal activity of hydroalcoholic extract of *A. tripedale* (50 mg/ml) against *C. krusei* by well diffusion assay after 48 h

Preliminary screening of the antifungal activity of hydroalcoholic extracts of *A. tripedale* was performed against *Candida* spp. using the disk and well diffusion assay. The results showed variation in the antifungal properties of hydroalcoholic extract of *A. tripedale* (Table 2). The extract showed strong activity (inhibition zone ⩾ 20 mm), moderate activity (inhibition zone < 12-20 mm), and no inhibition (zone < 12 mm). Fluconazole, a known antifungal antibiotic, as a positive control significantly inhibited the growth of *Candida *spp*. *(Figure 2). Based on the available evidence, the major effective antifungal activity by the hydroa-lcoholic extract was achieved against *C. albicans *(Figure 3). The hydroalcoholic extract of *A. tripedale *inhibited the growth of *C. parapsilosis* by well diffusion assay and *C. krusei* by disk diffusion assay in a dose-dependent manner (Figures 4, 5). 

**Table 1:** Phytocomponents identified in the hydroalcoholic extract of *A. tripedale* by gas chromatography and mass spectroscopy

**S.NO.**	**ID**	**RT**	**Area%**	**CAS**	**Molecular formula**	**Molecular weight g/mol**
1	2-Pentene, 2-methyl	3.133	0.2	625-27-4	CH_3_CH_2_CH=C(CH_3_)_2_	84.16
2	Furan, 2,4-dimethyl-	7.624	0.18	3710-43-8	C_6_H_8_O	96.1271
3	Ethane, 1,1,2,2-tetrachloro-	9.324	0.72	79-34-5	C_2_H_2_Cl_4_	167.8493
4	Benzaldehyde	11.435	0.19	100-52-7	C_7_H_6_O	106.1219
5	2,4 HEPTADIENAL	11.801	1.70	4313-03-5	C_7_H_10_O	110.1537
6	Nonanal	13.924	0.5	124-19-6	C_9_H_18_O	142.2386
7	Benzeneacetaldehyde	14.193	0.42	122-78-1	C_8_H_8_O	120.1485
8	2-Fluorophenylhydrazine	14.908	0.13	2368-80-1	C_6_H_7_FN_2_	126.1316
9	Octanoic Acid	15.441	0.35	124-07-2	C_16_H_30_O_4_Sn	405.117
10	Benzoic acid	1.654	0.24	65-85-0	C_7_H_6_O_2_	122.1224
11	Pyridine, 3-(phenylazo)-	17.581	0.18	2569-55-3	C_11_H_9_N_3_	183.21
12	4-Pyridinamine, N-methyl-N,3-dinitro-	18.73	0.14	104503-82-4	C_6_H_6_N_4_O_4_	198.13624
13	3-Methyl-2,3-dihydro-benzofuran	18.347	0.34	13524-73-7	C_9_H_10_O	134.1751
14	1-Carboxymethyl-2(1H)-pyridone	19.440	0.29	56546-36-2	C_7_H_7_NO_3_	153.1354
15	.alpha.-(Aminomethylene)glutaconicanhydride	20.12	0.15	67598-07-6	C_6_H_5_NO_3_	139.1088
16	2,4-Decadienal	20.093	0.43	2363-88-4	C_10_H_16_O	152.23344
17	2-Bromo-5-(hydroxymethyl)pyridine	20.178	0.12	122306-01-8	C_6_H_6_BrNO	188.02194
18	(E,Z,Z)-2,4,7-Tridecatrienal	20.310	0.67	1000314-35-6	C_13_H_20_O	192.3016
19	Hexadecane, 7,9-dimethyl-	20.836	0.74	21164-95-4	C18 H38	254.50
20	2,4-Decadienal	20.934	0.64	2363-88-4	C_10_H_16_O	152.2334
21	2-Methoxy-4-vinylphenol	21.763	0.6	7786-61-0	C_9_H_10_O_2_	150.1745
22	2-Propenoic acid, 3-phenyl-	24.910	0.41	621-82-9	C_9_H_8_O_2_	148.1586
23	2(4H)-Benzofuranone, 5,6,7,7a-tetrahydro-4,4,7a-trimethyl-, (R)-	28.687	0.25	17092-92-1	C_11_H_16_O_2_	180.24
24	Tetradecanal	29.265	0.26	124-25-4	C_11_H_20_O_2_	184.2753
25	1,1-Difluoro-2-methyl-3-ethyl cyclopropane	30.032	0.22	1000144-82-1	C6H10F2	120.140406
26	Cyclodecane	30.140	0.19	293-96-9	C10H20	140.27
27	Tetradecanoic acid	30.243	0.65	544-63-8	C_14_H_28_O_2_	228.37
28	2-Hexadecen-1-ol, 3,7,11,15-tetramethyl-, [R-[R*,R*-(E)]]-	30.306	4.67	150-86-7	C_20_H_40_O	296.531
29	Methyl .beta.-d-galactopyranoside	30.524	0.48	1000126-04-6	C_7_H_14_O_6_	194.18246
30	3-Pyridinamine, N-methyl-2-nitro-	30.655	0.15	32605-06-4	C_6_H_7_N_3_O_2_	153.1387
31	2-Pentadecanone, 6,10,14-trimethy	31.565	0.85	502-69-2	C_18_H_36_O	268.4778
32	Hexadecanoic acid	340380	6.91	57-10-3	C_16_H_32_O_2_	256.42
33	Phthalic acid, butyl undecyl ester	34529	0.92	1000308-91-2	C_23_H_36_O_4_	376.52954
34	Hexadecanoic acid, ethyl ester	34.586	0.41	628-97-7	C_18_H_36_O_2_	284.47724
35	Phthalic acid, propyl nonyl ester	36.641	0.41	1000309—06-4	C_20_H_30_O_4_	334.4498
36	Cyclohexanol, 1-methyl-4-(1-methylethyl)-	36.829	0.89	21129-27-1	C10H20O	156.27
37	Phthalic acid, isobutyl pent-2-en-4-yn-1-yl ester	45.761	1.56	1000315-45-6	C_17_H_18_O_4_	286.32242
38	1-Eicosanol	49.338	6.79	629-96-9	C_20_H_42_O	298.54688
39	Tetracosane	50.751	34.17	646-31-1	C_24_H_50_	338.6538
40	1,2-Dihydropyrido(3,2,1-kl)phenothiazin-3-one	54.236	4.94	69513-42-4	C_15_H_11_NOS	253.31894

**Table 2:** Antifungal activity of the *A. tripedale* in disk and well diffusion assay

**Zone of inhibition (mm)**													**Extract concentration** **(mg/mL)**	
***C. krusei***				***C. parapsilosis***				***C. albicans***						
**Well diffusion assay**		**Disk diffusion assay**		**Well diffusion assay**		**Disk diffusion assay**			**Well diffusion assay**		**Disk diffusion assay**			
**72**	**48**	**72**	**48**	**72**	**48**	**72**	**48**		**72**	**48**	**72**	**48**		
-	-	10±.0.25	17±0.11	28±0.34	28±0.28	-	-		10 ±0.28	30±0.17	10± 0.0	21±0.17	50	Hydro-alcoholic extract
-	-	-	5±.0.28	19±1.04	20±0.11	-	-		-	21±0.57	-	6±0.36	25	
-	-	-	5±.0.28	10±0.28	18±0.0	-	-		-	22±28	-	3±.28	12.5	
-	-	-	-	-	-	-	-		-	19±0.20	-	-	6.25	

## Discussion

Evidence suggests that fungal infections annually affect more than a billion people, and this rate is ever increasing. *Candida* spp. can be systemic or infect different parts of the body such as skin, nails, respiratory tract, urogenital system, and alimentary canal [20]. Although several species of *Candida* are potentially pathogenic in humans, *Candida albicans* is the most important cause of severe candidiasis [21]. Trizoles initially appear to be highly effective against fungal infections, but nowadays, increased resistance is being reported and azole resistant has instigated extensive research to evaluate the effect of antifungal agents from different sources, especially medicinal plants [19]. The most famous antifungal medicinal plants belong to Liliaceae family, where more reports are found on antifungal activity of *Allium* genus [22]. The antifungal properties of the *Allium* genus were mentioned in some studies. Shams-Ghahfarokhi et al. (2007) reported that aqueous extracts of *Allium cepa *and* Allium sativum* had antifungal activity against *Malassezia furfur, Candida *spp. and several strains of various dermatophyte species in a dose-dependent manner with the maximum of 100% at defined concentrations [23]. Another study by Amin and Kapadnis proved the antifungal activity of *Allium ascalonicum* against 23 fungal strains [24].

In this study, we examined the antifungal effect of hydroalcoholic extract of *A. tripedale* against different strains of *Candida* by disk and well diffusion assay. Our results revealed that the hydroalcoholic extract (50 mg/ml) had the greatest effect on *C. albicans*. However, it also had inhibitory effect against *C. parapsilosis* and *C. krusei*.

Based on the analysis conducted on the hydroalcoholic extract components using GC-MS method, 40 compounds were identified in this plant that had different properties. We found that tetracosane and other higher alkenes had antioxidant, antitumor, and antifungal properties, particularly against fungal spores and germination [25]. Tetradecanoic acid and eicosane had antioxidant and antimicrobial activities [26]. Hexadecanoic acid is known to have antioxidant and hypocholesterolemic properties and is a constituent of nematicides, pesticides, lubricants, antiandrogens, flavoring agents, hemolytics 5-alpha reductase inhibitors, antifeedants, and insect-repellents [27]. 

Benzoic acid derivatives possess antibacterial and antifungal properties. Phenazopyridine hydrochloride is a topical analgesic that relieves the irritative symptoms associated with urinary tract infection through acting on the mucosal lining of the urinary tract. This agent is compatible with antibiotics and relieves pain before the antibiotic begins to control the infection. Propionic acid is an important chemical commonly used as a raw material in different industries [28]. Propionic acid, the biopreservative produced by *Propionibacterium *spp., is capable of inhibiting the growth of molds, bacteria, and dairy-spoilage yeasts such as *Zygosaccharomyces bailii *and *Candida *spp. [29]. Phenolic compounds, esters, alkanes, aldehydes, alkenes, and ketones are the major volatile compounds, which have anti-inflammatory, antiarthritic, antidiabetic, antiulcer, hypolipidemic, antiatherosclerotic, anti-HIV, and cytotoxic activities [30]. Based on the results of the present study, hydroalcoholic extract of *A. tripedale *had a significant inhibitory effect against the growth of various strains of *Candida*. In sum, it seems that *A. tripedale* is a major source of anti-fungal compounds, which can be applied for the treatment of infectious diseases.

## Conclusion

This is the first report on the GC-MS analysis of *A. tripedale*. It can be concluded that *A. tripedale *contains various important bioactive compounds. Therefore, it is recommended as a plant of phytochemical and pharmaceutical importance. Further studies are required to isolate the active ingredients of the extract and elucidate its mechanism of action in various diseases.
